# Lumbar Lateral Dislocation Fracture With Progressive Worsening of Trunk Tilt in Pisa Syndrome Associated With Parkinson′s Disease

**DOI:** 10.1155/cro/3774288

**Published:** 2026-01-02

**Authors:** Kazuki Fujimoto, Narumi Maki, Daisuke Hashiba, Toshifumi Maeyama, Haruki Ito, Ryosuke Nakagawa, Tetsuhiro Ishikawa, Hajime Arai, Seiji Ohtori

**Affiliations:** ^1^ Department of Orthopedic Surgery, National Kohnodai Medical Center, Japan Institute for Health Security, Ichikawa City, Chiba, Japan; ^2^ Department of Orthopedic Surgery, Graduate School of Medicine, Chiba University, Chiba City, Chiba, Japan, chiba-u.ac.jp; ^3^ Department of Orthopedic Surgery, Sanmu Medical Center, Sanmu City, Chiba, Japan, sanmu-mc.jp

**Keywords:** lumbar fracture, Parkinson′s disease, Pisa syndrome, trunk tilt

## Abstract

**Introduction:**

Parkinson′s disease rarely presents with Pisa syndrome, for which a patient may require corrective surgery because of trunk tilt. Herein, we report a case of lumbar dislocation fracture caused by minor trauma. The patient′s trunk tilt progressed, and neurological symptoms worsened, requiring surgical treatment.

**Case Report:**

A 73‐year‐old man with Parkinson′s disease fell and gradually developed lower back pain, lower limb pain, and paralysis. Single‐segment interbody fusion at the dislocated intervertebral level temporarily improved neurological symptoms. However, 6 months after surgery, the implant had broken, and the trunk tilt had worsened. Salvage corrective surgery involving long‐range fixation improved trunk tilt. The patient recovered independence in activities of daily living.

**Conclusion:**

In Parkinson′s disease, minor trauma can cause lumbar dislocation fractures, which may worsen Pisa syndrome. Short‐segment fixation cannot control deformity progression; therefore, long‐range fusion should be considered during the initial surgery.

## 1. Introduction

With an aging society, the incidence of Parkinson′s disease (PD) has increased [1]. Among the postural abnormalities of PD, Pisa syndrome presents with a lateral flexion posture without spinal or vertebral deformities. Although various effective therapeutic interventions have been reported [2], corrective fusion surgery may still be required in some cases [3–6]. No reports exist of vertebral dislocation fractures in Pisa syndrome. Herein, we report a case of Pisa syndrome in which minor trauma resulted in a lumbar lateral dislocation fracture with lower limb paralysis. In this patient, single‐level posterior fixation failed soon after surgery. However, salvage corrective surgery involving long‐range fixation from T3 to the pelvis showed good progress 1 year after surgery.

## 2. Case Presentation

The patient was a 73‐year‐old man who had been diagnosed with PD 5 years earlier with bradykinesia and postural instability that manifested as abnormal gait posture. He was treated with oral medication, beginning with a dopamine agonist, the dosage of which was gradually increased. The most recent treatment consisted of levodopa (750 mg/day) and a dopamine agonist (3 mg/day). Then, 4 months before being examined by our institution, the patient′s trunk tilting to the right became more noticeable. Then, 3 months before visiting our institution, he developed lower back pain after falling at home. Then, 1 month before presenting to our institution, the dopamine agonist, which may have exacerbated his abnormal posture, was discontinued and replaced with a rotigotine transdermal patch (4.5 mg/day). Then, 2 weeks before presenting to our institution, he developed pain and muscle weakness in his right lower limbs, which gradually worsened, despite taking oral painkillers. He started having difficulty walking 1 week before presenting to our institution.

During his first visit to the outpatient clinic, he complained of pain and numbness from the right hip to the front of the thigh. Physical examination revealed paralysis of the proximal muscles of the right lower limb (right iliopsoas and manual muscle test 2–3).

Radiography and magnetic resonance imaging revealed focal scoliosis and severe spinal stenosis at the L2–3 level (Figure 1). The patient was immediately admitted to our hospital. Myelography and computed tomography revealed a right lateral dislocation fracture of L2 with a rightward tilt of the trunk and spinal stenosis at the L2–3 level (Figures 2 and 3). Bone mineral density (*T*‐score) assessed using dual‐energy X‐ray absorptiometry (Hologic Discovery) was 0.8 at the lumbar spine and −0.8 at the femoral neck, indicating no osteoporosis.

Figure 1Initial radiographs and magnetic resonance images.The radiographs show focal scoliosis at the L2–3 level (a [arrowhead] and b). T2‐weighted magnetic resonance images at the L2–3 level show focal scoliosis (c) and severe spinal stenosis (d).

**Figure:**
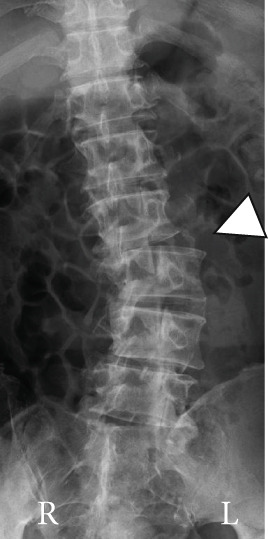
(a)

**Figure:**
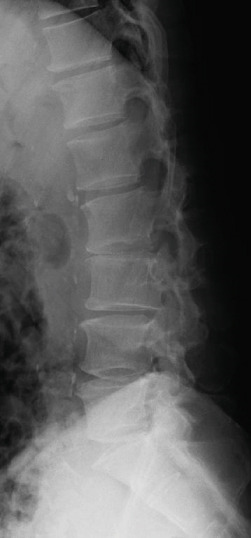
(b)

**Figure:**
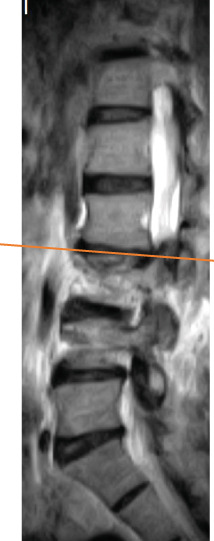
(c)

**Figure:**
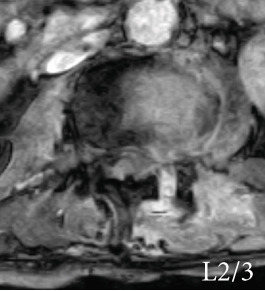
(d)

Figure 2Myelography before the initial surgery. Myelography shows spinal canal stenosis at the L2–3 level (a, b) and spinal instability in left (c) and right (d) flexion.

**Figure:**
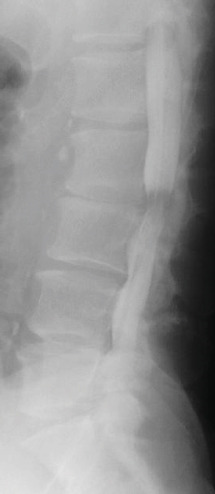
(a)

**Figure:**
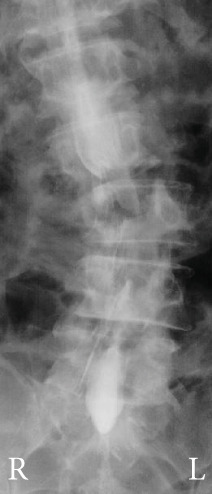
(b)

**Figure:**
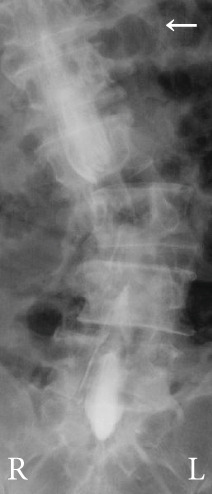
(c)

**Figure:**
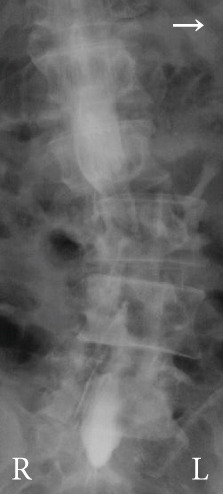
(d)

Figure 3Computed tomography myelography before the initial surgery. Computed tomography (CT) images show a right‐sided dislocation fracture of the right inferior articular process of L2 (arrowhead) and right upper articular process of L3 protruding into the intervertebral foramen (arrow) with a rightward tilt of the trunk and spinal stenosis at the L2–3 level.

**Figure:**
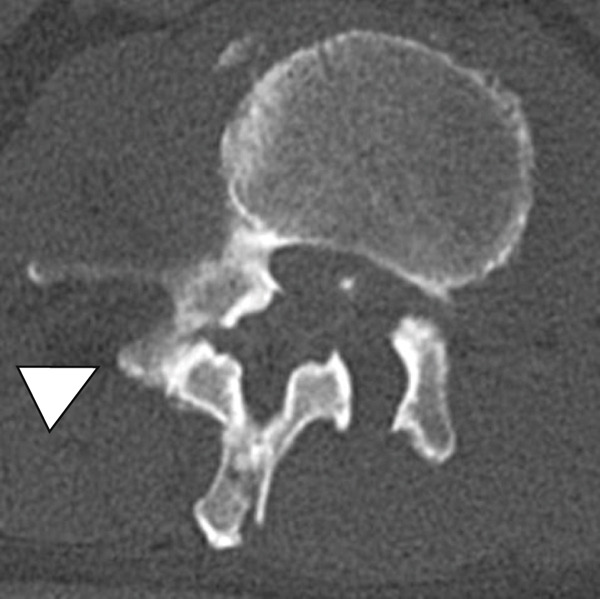
(a)

**Figure:**
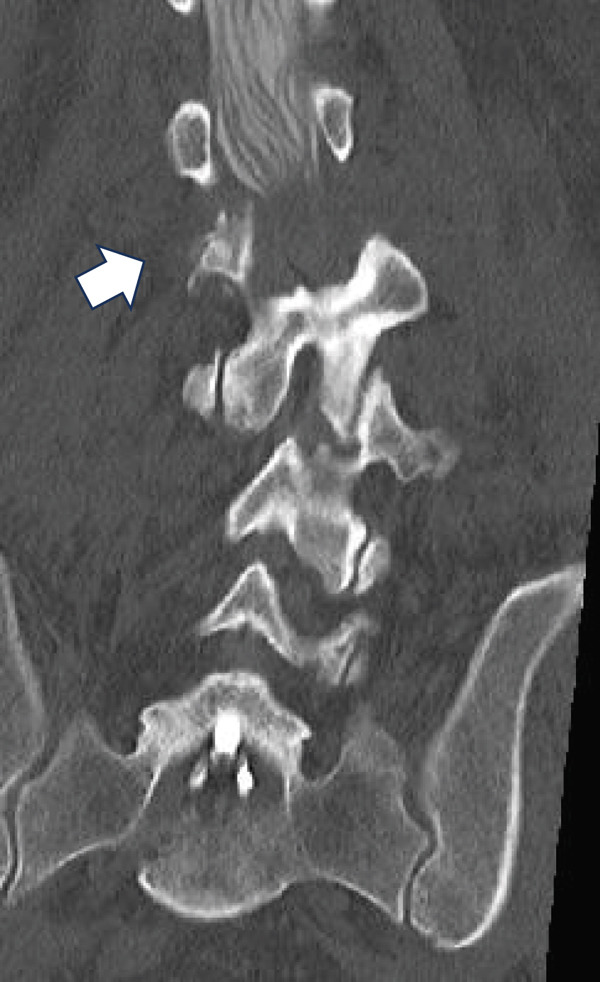
(b)

We considered his lower limb paralysis. Therefore, surgery was expedited, and a single posterior intervertebral interbody fusion of L2–3 was performed. Intraoperative findings showed a right L2 inferior articular process fracture and protrusion of the right L3 superior articular process into the L2–3 intervertebral foramen, with compression of the right L2 nerve root.

During surgery, bilateral facetectomy was performed, intervertebral cages were placed posteriorly on both sides, pedicle screws were placed, and rod fixation was performed. Placement of the cages on both vertebral body margins was attempted; however, on the right side, the cage was placed too close to the margin (Figure 4a), revealing cage dislodgement on a radiograph taken 1 week after surgery. Nevertheless, the patient′s lower limb symptoms gradually improved after surgery (Figure 4b,c).

Figure 4Follow‐up radiographs after initial surgery. Radiographs of the lumbar spine after initial surgery. Posterior lumbar interbody fusion with bilateral interbody cages, pedicle screws, and rods was performed. Compared with the immediate postoperative radiograph (a), the right‐sided cage shows dislodgement at 1 week after surgery (b, c; arrows in a and b). Radiographs of the whole spine 8 months after the initial surgery show trunk tilt exacerbation (d) and kyphosis at the cranial side of the fixed vertebrae (e). A broken pedicle screw was inserted in the right L2 pedicle (f; arrowhead in d).

**Figure:**
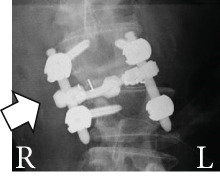
(a)

**Figure:**
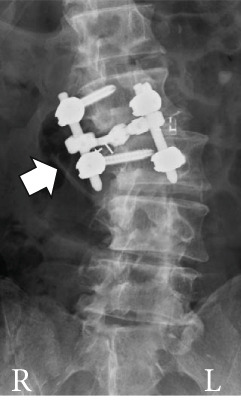
(b)

**Figure:**
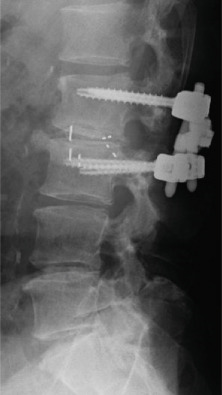
(c)

**Figure:**
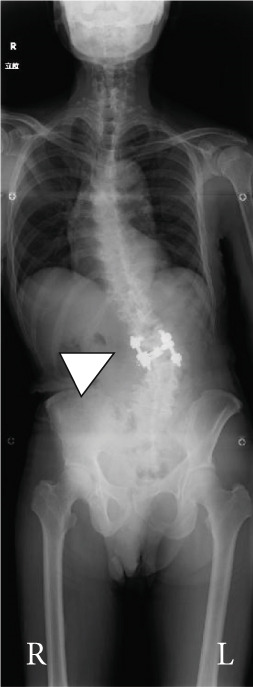
(d)

**Figure:**
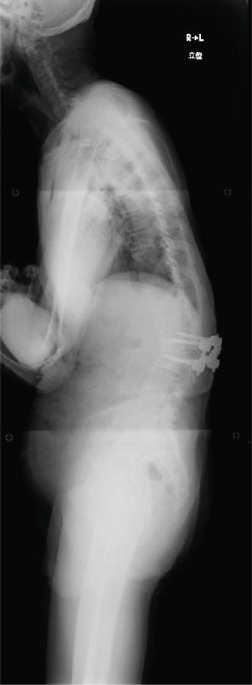
(e)

**Figure:**
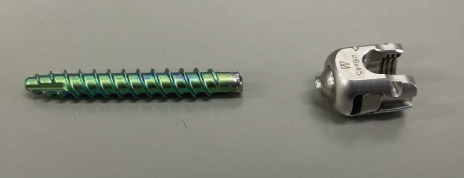
(f)

He was able to walk 2 months after surgery, and his postoperative course was uneventful. However, during an outpatient follow‐up approximately 6 months after surgery, the patient showed progression of trunk tilt toward the right side and worsening back pain, although he did not complain of lower limb pain. Approximately 8 months after surgery, his symptoms worsened, and the patient began to have trouble walking because of back pain. Therefore, another surgery was planned (Figure 4d,e).

A two‐stage surgery was performed. Stage 1 involved the removal of the posterior implant and anterior fixation of L1–5 using anterior intervertebral cages. Intraoperative findings revealed that a screw inserted into the right pedicle of L2 had broken (Figure 4f). Then, 1 week after Stage 1 surgery, Stage 2 was performed and involved posterior deformity correction. Fixation from T3 to S2, including facetectomy at all lumbar vertebrae, was performed using alar iliac screws (T3 was the upper end and S2 was the lower end).

Balance in the coronal and sagittal planes improved postoperatively. The sagittal vertical axis decreased from 134 mm before surgery to 24 mm after surgery, and in the coronal plane, the C7‐center sacral vertical line decreased from 58 mm on the right to 15 mm on the left.

Lumbar lordosis was −5° before surgery and 29° after surgery. Thoracic kyphosis was 25° before surgery and 38° after surgery. No change occurred in pelvic incidence (32° before and after surgery; Figure 5). The patient′s lateral tilt improved, and he was able to walk independently 2 months postoperatively. Despite referred scapular pain due to proximal junctional failure (PJF) of the T2 vertebral body 6 months after surgery, the patient had a good 1‐year postoperative course, with pain gradually improving (Figures 6a, 6b, and 6c). He was independent in activities of daily living. Spinopelvic parameters before and after surgery are shown in Table 1. Teriparatide therapy was initiated after PJF onset, and follow‐up computed tomography images, obtained 2 years after surgery, showed bone union of the T2 PJF without any change in alignment or implant failure (Figure 6d).

Figure 5Radiographs after the second salvage surgery.Radiographs of the whole spine after the second salvage surgery show improvement in trunk tilt and kyphosis. Coronal (a) and sagittal (b) views.

**Figure:**
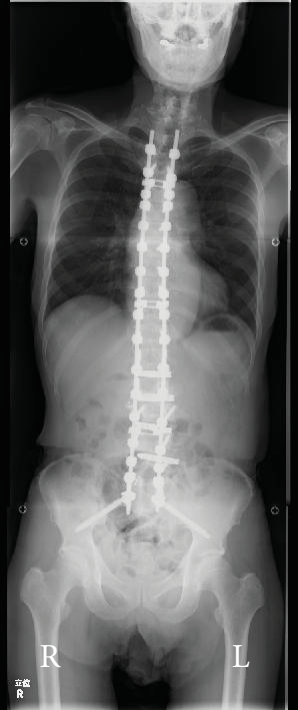
(a)

**Figure:**
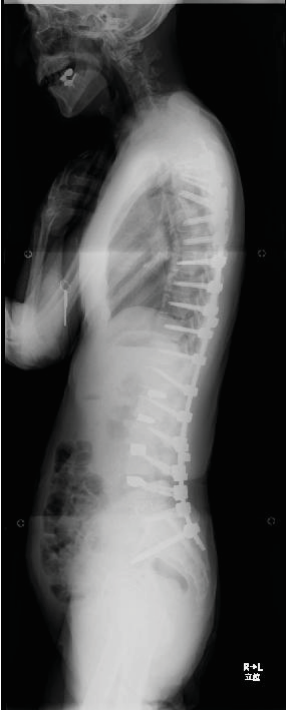
(b)

Figure 6Follow‐up radiographs and computed tomography images after the second salvage surgery. Radiographs (a, b) and computed tomography (CT) images (c) of the whole spine 1 year after the second salvage surgery show a proximal junctional fracture of the T2 vertebrae (arrowhead in c). The CT image obtained 2 years after the second surgery shows bone union of the T2 vertebrae (arrowhead in d).

**Figure:**
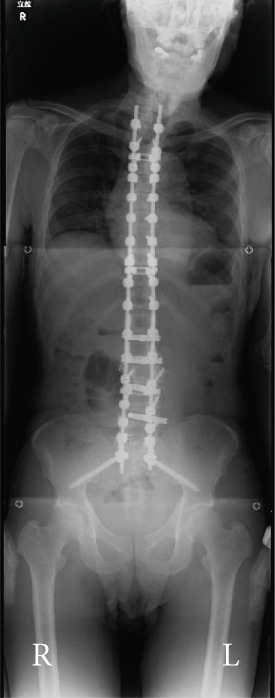
(a)

**Figure:**
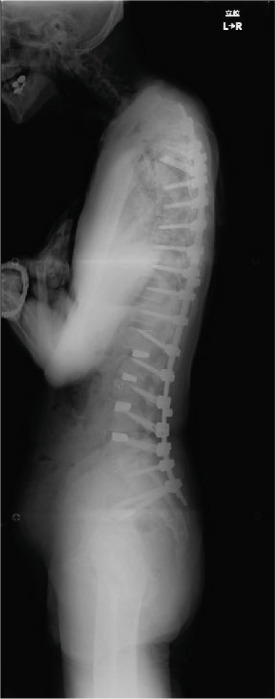
(b)

**Figure:**
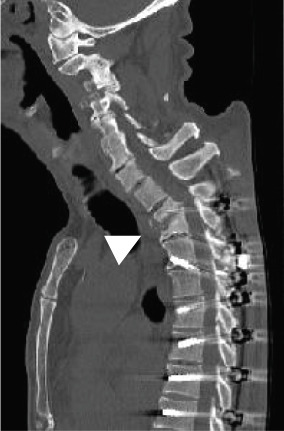
(c)

**Figure:**
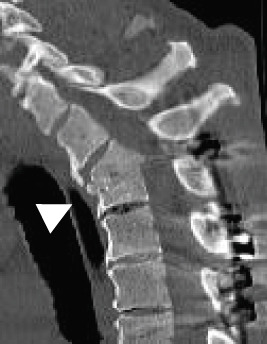
(d)

**Table 1:** Spinopelvic parameters before and after surgery.

	**Before surgery**	**After surgery**	**1 year post-surgery**
C7‐plumb (SVA) (mm)	134	24	36
Thoracic kyphosis (deg.)	25	38	39
Lumbar lordosis (deg.)	−5	29	29
Pelvic tilt (deg.)	26	16	16
Sacral slope (deg.)	6	15	18
Pelvic incidence (deg.)	32	32	32
Cobb angle (deg.)	(T3‐L1) 32 (L1‐5) 36	0	0
C7‐plumb (frontal) (mm)	Right 58	Left 15	Left 15

Abbreviations: deg., degrees; SVA, sagittal vertical axis.

## 3. Discussion

In patients with Pisa syndrome associated with PD, minor trauma can cause lateral lumbar dislocation and lead to severe neurological symptoms. In such cases, short‐segment fixation is insufficient to reduce the progression of lateral tilt. Reconstructive surgery with long‐range fixation is required. Patients with PD present with correctable scoliosis at a frequency of 8% [7]. Among these patients, a scoliosis angle ≥ 10^°^ is considered indicative of Pisa syndrome [8], which has a prevalence of approximately 2% among patients with PD [9]. Surgery is often required to improve spinal balance that is resistant to conservative treatment [3]. In addition, patients with PD are prone to falls due to abnormal body axis posture and gait freezing [10], and they are more likely to develop compression fractures due to minor trauma than patients without PD [11]. However, to the best of our knowledge, no reports of vertebral dislocation fractures in Pisa syndrome have been reported to date. Therefore, this case is the first report of a patient with PD who experienced minor trauma that resulted in a lumbar lateral dislocation fracture, followed by the progression of Pisa syndrome and severe neurological symptoms.

Furthermore, single‐segment interbody fusion was performed at the dislocated intervertebral level. However, the implant had become dislocated 6 months after surgery, necessitating long‐segment fixation. Spinal surgery for PD has a high complication rate, a high reoperation rate, and a low bone union rate [12–16]. Long‐segment fixation, in particular, has a higher failure rate than short‐segment fixation, and therefore, surgical procedures must be carefully considered [17]. Since the patient′s primary complaint was neurological symptoms presenting as paralysis, urgent decompression was the primary goal. Therefore, single‐level posterior lumbar interbody fixation was performed, which minimizes surgical invasiveness and has a low failure rate. However, early postoperative cage dislodgement and progressive trunk tilt were observed, indicating that short‐segment fixation was insufficient to support the persistent asymmetric loading of Pisa syndrome, which presents high instability, like dislocation fractures. Short‐segment fixation is preferred for patients with PD whenever possible because multilevel fusion has a higher complication rate and a worse prognosis [18]. However, as in the present case, insufficient fixation force may lead to implant failure and progressive trunk tilt. When performed by an experienced surgeon familiar with the unique needs of patients with PD, long‐segment fixation can lead to significant improvements in pain, walking ability, and spinal balance [3, 4]. Successful outcomes depend on proper patient selection, multidisciplinary management, and achieving proper sagittal alignment through vigorous instrumentation [5, 6]. However, no consensus exists regarding the appropriate extent of fixation. Reports suggest that extending fixation to at least the mid‐thoracic spine to prevent PJF and to the sacrum to prevent distal junction degeneration is beneficial for patients at risk of sagittal decompensation and distal junctional degeneration [19], such as those with PD.

In this patient, salvage surgery was performed for fusion of T3 to the pelvis, improving coronal and sagittal alignment. Although the patient experienced a fracture at the proximal adjacent vertebra 1 year after surgery, bony union ultimately resulted in a good outcome. PJF in patients after long‐segment fixation is one of the most concerning complications. The causes of PJF are diverse, including high body mass index, inadequate postoperative sagittal balance, advanced age, pelvic instrumentation, and osteoporosis [20], but there have been no adequate prospective studies [21]. Furthermore, in PD, as the disease progresses, kyphosis of the thoracic or thoracolumbar spine progresses, resulting in a stooped posture [22]. Therefore, adjacent segment disease due to kyphotic deformity tends to occur at the intervertebral level adjacent to the upper end of the fusion, and it is closely associated with the development of PJF. Accordingly, when performing long‐segment fixation, careful consideration is required for selecting the upper instrumented vertebra and restoring sagittal balance [19]. Perioperative administration of teriparatide to patients with osteoporosis after spinal deformity surgery has been reported to effectively reduce the incidence of PJF and postoperative lower back pain [23, 24]. Moreover, more than 6 months of administration of teriparatide after lumbar fusion surgery has been reported to be effective in promoting bone union [25].

In this patient, there was no surgical overcorrection or low bone mineral density, but PJF in the stress‐concentrated vertebrae developed as PD progressed. Teriparatide administration after PJF onset may have contributed to the bony union of the PJF.

Pisa syndrome in PD can lead to lumbar dislocation fractures even after minor trauma. Surgical treatment is required in the presence of neurological symptoms. Short‐segment fixation cannot control deformity progression; therefore, long‐segment fixation should be considered during the initial surgery. Perioperative teriparatide administration is recommended for the prevention of PJF.

Similar outcomes may occur in other cases of spinal trauma in PD. However, this observation is based on a single case report, and long‐term follow‐up is required.

## Ethics Statement

The patient was informed that their data would be submitted for publication and provided consent.

## Consent

Informed consent was obtained from the patient.

## Conflicts of Interest

The authors declare no conflicts of interest.

## Funding

The authors did not receive support from any organization for the submitted work.

## Data Availability

The data that support the findings of this study are available on request from the corresponding author.
